# Response of rhizosphere soil microbial to *Deyeuxia angustifolia* encroaching in two different vegetation communities in alpine tundra

**DOI:** 10.1038/srep43150

**Published:** 2017-02-21

**Authors:** Lin Li, Ming Xing, Jiangwei Lv, Xiaolong Wang, Xia Chen

**Affiliations:** 1 National & Local United Engineering Laboratory for Chinese Herbal Medicine Breeding and Cultivation, Jilin University, Changchun 130112, China; 2 School of Life Science, Jilin University, Changchun 130012, China; 3 Huhhot Vocational college, Huhht, Inner Mongolia 010051, China

## Abstract

*Deyeuxia angustifolia* (Komarov) Y. L Chang is an herb species originating from the birch forests in the Changbai Mountain. Recently, this species has been found encroaching into large areas in the western slopes of the alpine tundra in the Changbai Mountain, threatening the tundra ecosystem. In this study, we systematically assessed the response of the rhizosphere soil microbial to *D. angustifolia* encroaching in alpine tundra by conducting experiments for two vegetation types (shrubs and herbs) by real-time PCR and Illumina Miseq sequencing methods. The treatments consisted of *D. angustifolia* sites (DA), native sites (NS, NH) and encroaching sites (ES, EH). Our results show that (1) Rhizosphere soil properties of the alpine tundra were significantly impacted by *D. angustifolia* encroaching; microbial nutrient cycling and soil bacterial communities were shaped to be suitable for *D. angustifolia* growth; (2) The two vegetation community rhizosphere soils responded differently to *D. angustifolia* encroaching; (3) By encroaching into both vegetation communities, *D. angustifolia* could effectively replace the native species by establishing positive plant-soil feedback. The strong adaptation and assimilative capacity contributed to *D. angustifolia* encroaching in the alpine tundra. Our research indicates that *D. angustifolia* significantly impacts the rhizosphere soil microbial of the alpine tundra.

The alpine tundra of the Changbai Mountain is one of the two rare alpine tundra distributions in China. Alpine habitats are characterized by harsh conditions such as strong winds, short growing seasons, high solar radiation, and low temperatures; these factors result in a relatively low risk of plant invasions^1^. However, studies have shown that *D. angustifolia* has been inhabiting the alpine tundra of the Changbai Mountain since the 1990s^2^, although it is a herbaceous species which usually lives in the birch forest zone of the Changbai Mountain^3^. Encroachment of *D. angustifolia* is probably a result of a typhoon in 1986, which caused significant canopy gaps^4^ and of increased nitrogen deposition^5^. The *D. angustifolia* encroachment into alpine tundra is similar to colonization by invasive plants. In the case of *D. angustifolia*, the first occurrence has been reported more than 30 years ago, however, research on this species is limited to aboveground studies evaluating changes of vegetation cover and plant community diversity^6^,^7^. Studies evaluating changes in soil biodiversity and below-ground community structures are scarce.

Below-ground biodiversity is a key resource for maintaining the functioning of ecosystems^8^,^9^. A large proportion of the biodiversity within terrestrial ecosystems is hidden below ground in soils, and the impact of altering its diversity and composition on the performance of ecosystems is still poorly understood^10^,^11^,^12^. Soil communities are extremely complex and diverse^12^, and microorganisms play critical roles in regulating soil fertility, plant health, plant community dynamics, and nutrient cycling^13^,^14^. In turn, soil microbial communities are considerably impacted by various factors such as nutrient amounts, moisture, or temperature^14^,^15^,^16^. It is also known that plant species can alter soil properties, mainly in the area around the rhizosphere, impact soil microbial communities, and thereby influence plants nearby^17^,^18^. These fungi associate with roots of the host plant, increasing absorptive surface area, allowing for enhanced uptake of essential nutrients and increasing water availability and drought tolerance^19^. The biopolymers in fungal mycelia represent an important source of both carbon and nitrogen^20^. Fungal abundance may play a critical role in the continued success of non-native species invisibility^21^. Thriving of invasive plants in new environments have been implicated in changes in the soil microbial community, root distribution in the soil, and other soil properties^22^,^23^. These changes subsequently influence important biogeochemical functions such as nitrogen cycling^24^,^25^,^26^. An invasive species may influence soil nitrogen cycling through direct interactions with the soil microbial community or through its effects on soil nitrogen itself^26^. For example, the abundance of ammonia-oxidizing bacteria (AOB) and ammonia oxidizing archaea (AOA) may be directly influenced by organic carbon, total nitrogen, and availability of ammonium^27^,^28^, N enrichment is shown to change the abundance of functional N-fixation genes (*nifH*)^29^,^30^. In turn, nitrogen fixing bacteria population sizes could increase N input in rhizosphere was conducive to plant invasion^31^.

Invasive species may establish stronger positive plant-soil feedback mechanisms by altering nearby soil chemical properties^32^,^33^, by changing soil microbial communities and microbial functions^30^,^34^,^35^, by leaving their belowground natural enemies behind^36^,^37^, or by decreasing native diversity and richness^38^,^39^. In some cases, native plants do not adapt to the new soil environment. In particular, herbaceous invasive plants are able to take advantage of growing in soils with high N concentrations (atmospheric N deposition or nitrogen input)^40^,^41^ or might impact biological nitrogen fixation^29^. But not all invasion plants have the same phenomenon, some have negative or neutrality feedback^42^,^43^.

The alpine tundra in the western slope of the Changbai Mountain has two main plant types: herbaceous perennials and low woody shrubs. Native and invasive species may differently affect soil environments. The rhizosphere usually contains a diverse microbial community^44^,^45^ which is directly influenced by plant root activities^46^. In order to fully understand the effect of *D. angustifolia* on the alpine tundra soil, we evaluated the microbial ecology of rhizosphere soil from *D. angustifolia* and different native companion plants. We tested the following hypotheses: (1) plant communities and soil microbial communities respond differently to *D. angustifolia* encroachment; (2) *D. angustifolia* changes soil properties, nutrient dynamics, and community structure, thereby enabling further expansion of its habitat.

To test these hypotheses, we analyzed rhizosphere soil from *D. angustifolia* in its independent community, native species community and encroachment community in regards to soil properties, microbial N cycling, fungal biomass and soil microbial community composition. We then evaluated the variation of native above-ground community diversity and predicted biotic interactions between two plant types in the community. Finally, using observational data, we draw conclusions about the rhizosphere responses to *D. angustifolia* encroachment alpine tundra ecosystems.

## Results

### Changes in soil nutrient dynamics plant communities associated with *D. angustifolia* colonization

**Figure 1:**
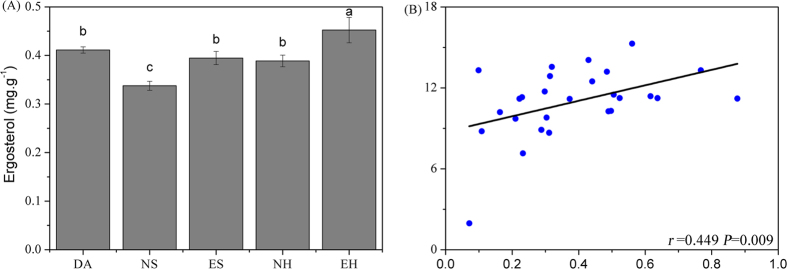
(**A**) Ergosterol (mg.g^−1^) in soil attached rhizosphere (mean ± 1 SE, n = 5) for five treatments of *D. angustifolia*, native and encroachment with two vegetation types. (**B**) Relationships between fungal biomass and TN.

Soil parameters varied across the five sampling sites. Sites with *D. angustifolia* (DA) were generally less nutrient rich in terms of nutrient pools (TN, TOC, TK), moisture and soil enzymatic activities (except ACP) compared with the other four sites (Table 1). Comparing native (NS, NH) and encroachment sites (ES, EH), ES sites contained a higher amounts of nutrients (*P* < 0.05), with the exception of total potassium (TK) than NS; EH exhibited slightly TN and TC, but these values were not significantly different compared to NH (*P* > 0.05). The sites ES and EH contained more available nutrients (AN, AK, AP), MBC, MBN and had higher enzymatic activities compared to native plots (NS and NH). These results indicate that soil available nutrients were more sensitive than other nutrient forms to *D. angustifolia* encroachment. Extractable nitrogen (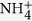, 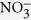) in DA and ES sites was significantly lower than in NS sites, while there were no differences between NH and EH. In contrast, DA soil samples had higher pH values than NS and lower values than NH, but pH values in EH samples was similar to DA and in ES was higher than in NS. Comparing the rangeability of soil parameters in plant sites encroached by *D. angustifolia* (Fig. S1) suggests that total nutrients (TP, TK), available nutrients (AN, AK, AP), and soil enzymatic activities (sucrase, acid phosphatase and catalase) in herbaceous plant sites were more sensitive to encroachment than in shrub plant sites, in contrast to other nutrients. To investigate the cause of these changes in soil properties, we performed correlation analysis between soil properties and soil enzymatic activities (Table S3). Soil enzymatic activities were significantly positively correlated with total nutrients (TN, TOC, TP), moisture and C/N, while acid phosphatase was only significantly correlated with TP. Soil enzyme activities were also positively correlated with soil available nutrients (AN, AP, AK). As soil fungi are also involved in soil nutrient cycling, we measured fungal biomass (Fig. 1A), observing lowest biomass values in NS and significantly higher values in encroaching sites (ES, EH) than in native sites (NS, NH). The values of fungal biomass in DA, NH and ES were not significantly different. Correlation analyses (Fig. 1B) showed that fungal biomass was significantly (*P* < 0.05) associated with TN.

**Figure 2:**
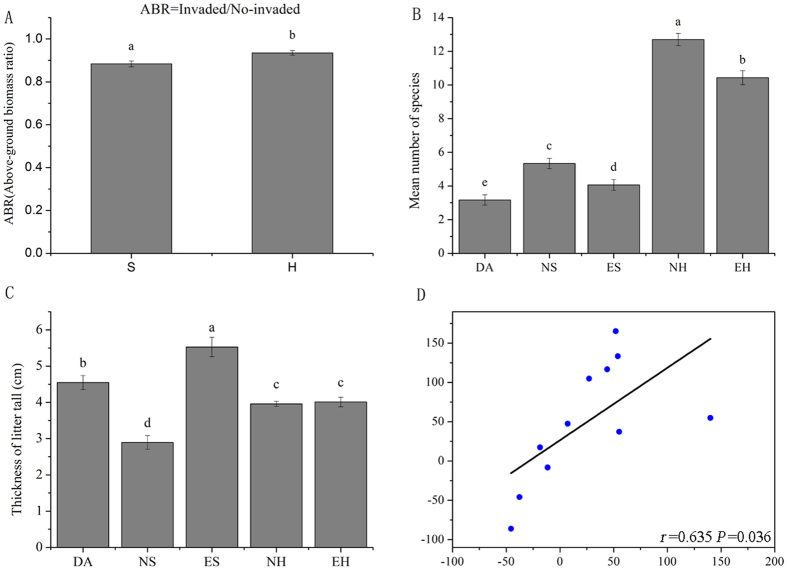
(**A**) The ratio of above-ground biomass (mean ± 1 SE, n = 2) between the encroaching plant, *D. angustifolia* and the native plant, under treatments of shrub and herbaceous vegetation types. (**B**) Species richness index (mean ± 1 SE, n = 5) for five treatments of *D. angustifolia*, native and encroachment with two vegetation types. (**C**) Thickness of litter tall (mean ± 1 SE, n = 5) five treatments. (**D**) Relationships between thickness of litter tall density and TOC. The lower case letters ‘a’, ‘b’, ‘c’ and ‘d’ indicate contrasts that are significantly different (*P* < 0.05) among different treatment.

Above-ground biomass significantly decreased from native to encroachment types, showing a similar trend for the two plant types (Fig. 2A). The degree of above-ground biomass was higher in the shrub community, and species richness was lower in invaded plots (ES, EH) than in native plots (NS, NH) (Fig. 2B). However, leaf litter thickness was higher after *D. angustifolia* encroaching (Fig. 2C), possibly causing changes in soil nutrient dynamics. Correlation analysis revealed that TOC and leaf litter thickness were strongly correlated (Fig. 2D), suggesting that higher amounts of litter increase soil organic matter.

### Soil N-cycling functional groups change associated with *D. angustifolia* encroaching

**Figure 3:**
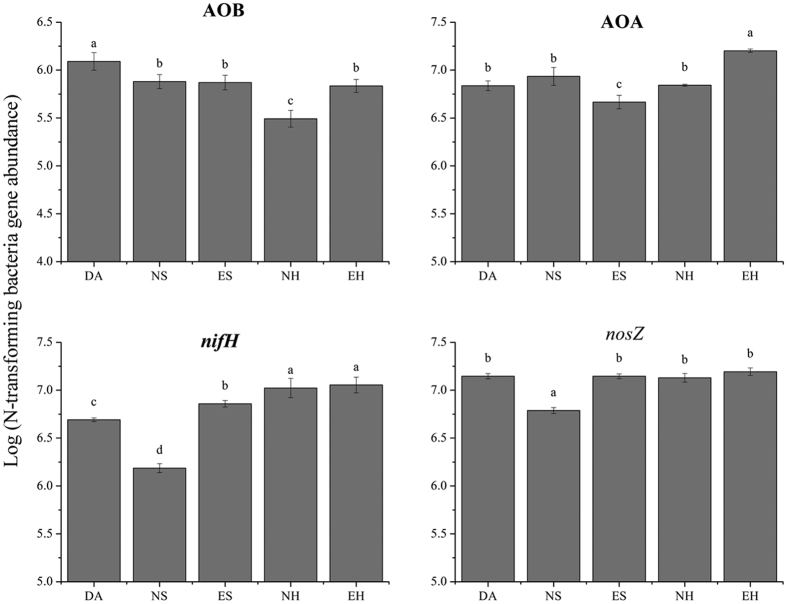
Real-time PCR quantification of the N-transforming function genes (*nifH*, archaeal *amoA*, bacterial *amoA* and *nosZ*) in the soil under *D. angustifolia* (DA), native shrub (NS), encroaching in shrub (ES), native herbaceous (NH) and encroaching in herbaceous (EH). The copy number of genes in each gram of dry soil was estimated based on the results of real-time PCR (copies per ng DNA). The standard curve of all these genes was >0.99. The lower case letters ‘a’, ‘b’, ‘c’ and ‘d’ indicate contrasts that are significantly different (*P* < 0.05) among different treatment. Each sample was measured in triplicate.

The abundance of N-cycling functional groups (bacterial *amoA*, archaeal *amoA, nosZ* and *nifH*) was assessed for five sampling sites (Fig. 3). *D. angustifolia* encroachment into herbaceous communities had no impact on soil *nifH* (N-fixation bacteria) and nosZ (denitrifying bacteria), but positively affected nitrobacteria (AOA, AOB) when NH was compared to EH. In the shrub soil sample, NS had the lowest abundance of *nosZ* and *nifH* genes, while in ES, abundance was significantly increased (*P* < 0.05) by *D. angustifolia* encroachment and compare to NH and EH have no such changed like NS and ES (Fig. 3). When the abundance of function gene was higher (*P* < 0.05) in DA than in native site (NS or NH), the function gene abundance in encroaching site (ES or EH) could be increased (Fig. 3, Fig. S2), such as AOB gene in NH and EH, *nifH* and *nosZ* genes in NS and ES.

Pearson correlation coefficients (*r*) between N cycling function genes abundance and soil properties revealed that nutrient function genes in all samples were significantly influenced by moisture and extractable nitrogen (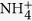, 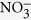) (Table S4). We calculated the correlation of N cycling function genes abundance and soil properties of shrub soil and herbaceous soil separately (Table S4). Function gene abundance was more susceptible to the effects of soil environment in herbaceous samples than in shrub samples. As abundance of AOB and *nifH* genes was not significantly correlated to any soil properties in the shrub samples (Table S4). Function gene abundance was mostly associated with total soil nutrients (TOC, TK, TP and C/N) in herbaceous soil samples and with soil available nutrients (AN, AK, 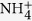 and MBC) in shrub soil samples.

### Soil microbial community change associated with *D. angustifolia* encroaching

A total of 1.8 million high quality 16S rDNA gene sequence reads were obtained from 27 samples. There were 27, 135 distinct OTUs (observations) across samples, with a total of 633, 077 sequences (counts) that were assigned to these OTUs. This observation refers to the number of distinct OTUs, whereas the count refers to the abundance of bacteria belonging to these OTUs in the samples. The average Good’s coverage for the bacterial data across 27 samples was 98.98% on average. Chao1, observed species, Shannon, Simpson, goods coverage and PD whole tree metrics were used to calculate alpha diversity (species diversity within the community) (Table S5). A non-parametric test showed differences among encroaching, native and *D. angustifolia* samples for alpha diversity (Table S5), with significantly lower richness in ES than in NS, probably due to the lowest biodiversity of *D. angustifolia* (Tables S5 and S6). Similar to shrub plant soil samples, biodiversity of EH was also decreased, albeit not significantly compared to NH. Among *D. angustifolia*, NS and NH were significantly different in chao1 and had good coverage (*P* ≤ 0.05), while encroaching plots had an alpha diversity similar to *D. angustifolia* (Tables S5 and S6). The OTU richness in the soil was not correlated with soil properties, except TOC (Fig. S3).

**Figure 4:**
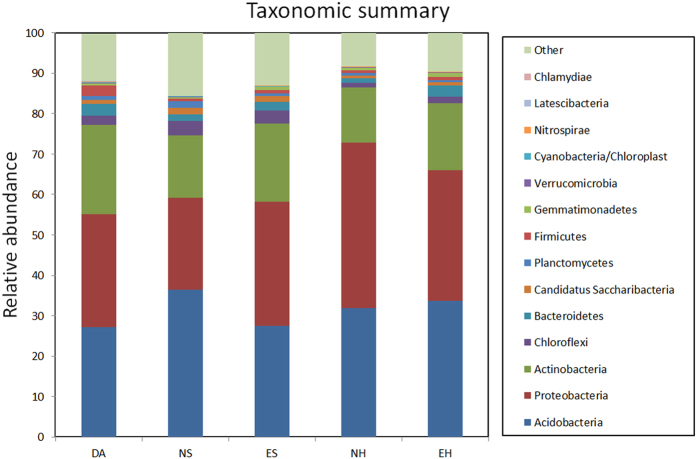
Taxonomic summary of the relative abundance of bacterial phyla in the *D. angustifolia* sites (DA), native shrub sites (NS), encroaching in shrub sites (ES), native herbaceous sites (NH) and encroaching in herbaceous sites (EH). The taxa are arranged as per total relative abundance across all samples, with the most abundant phyla at the bottom and the least abundant phyla at the top of the y-axis. Similarly, the phylum names in the legend are arranged from the most abundant at the top to the least abundant at the bottom.

Among the identified groups, *Acidobacteria* (31%) was the most dominant phylum, while *Proteobacteria* was the second most abundant phylum, accounting for 30% of all sequences; *Actinobacteria* were accounting for 17% (Fig. 4). Testing by ANOSIM revealed that three dominant bacteria phyla showed significantly different abundance in NS and ES, only one phyla showed significantly different abundance in NH and EH. Four dominant phyla were significantly different between *D. angustifolia* and native sites (NS, NH), while there was no difference after encroaching (Table S7). To evaluate the difference between soil microbial communities in native and encroaching sites, we contrasted the relative abundance at the genus level.

Ten of OTUs that showed significantly different abundances between NS and ES, abundances of *Devosia, Dokdonella, Gemmatimonas, Nakamurella, Phaselicystis, and Streptosporangium* were higher, while *Conexibacter* abundance was lower in ES than in NS (Table 2). Among them, the nitrogen-fixing microbes *Devosia* and *Gemmatimonas* showed relative abundances 6.2 and 2.0 times greater in encroaching than in native soil samples (Table 2). In the herbaceous soil samples, only two OTUs showed significantly different abundances between NH and EH samples.

**Figure 5:**
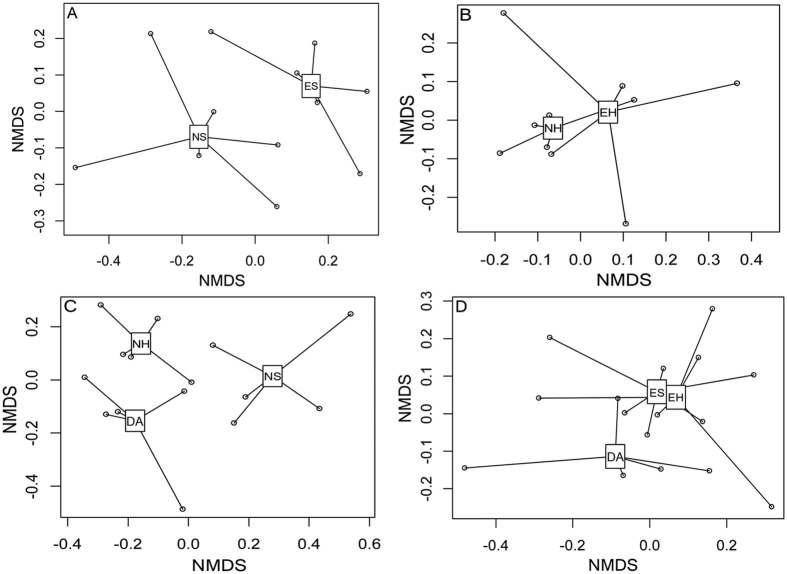
Non-metric multidimensional scaling (NMDS) ordination of microbial communities in native (NS, NH) and encroachment (ES, EH) and *D. angustifolia* sites (DA) five treatment, analyses NS vs ES, NH vs EH, NS vs NH vs DA and DA vs ES vs EH respectively. The difference of bacterial communities by anosim analysis (**A**) *R* = 0.33, *P* = 0.012; (**B**) *R* = 0.116, *P* = 0.112; (**C**) *R* = 0.488, *P* = 0.002; (**D**) *R* = 0.042, *P* = 0.265.

**Figure 6:**
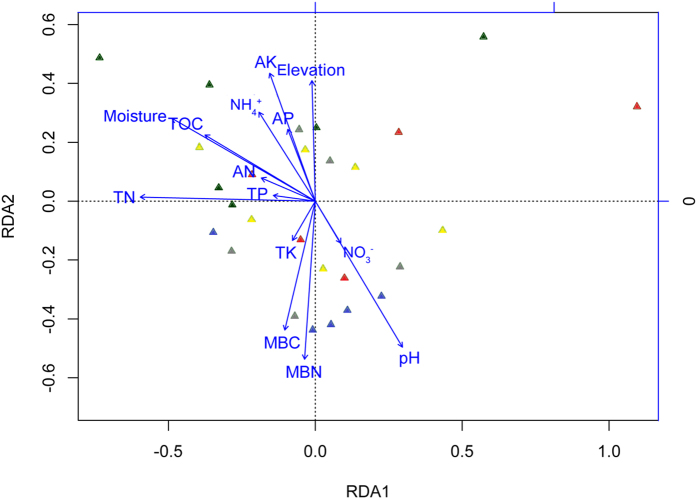
Redundancy analysis (RDA) triplots of 16S rRNA fingerprint patterns, showing the contribution of 14 environmental parameters to variability, 60% of total variation in the data can be explained. Arrows indicate environmental factors and their relative effects on microbial community structure. The red triangles indicate DA; green triangles indicate NS; yellow triangles indicate ES; blue triangles indicate NH and gray triangles indicate EH.

Non-metric multidimensional scaling (NMDS) ordination revealed that microbial communities were significantly different between NS and ES (*P* = 0.012), but no significant difference (*P* = 0.112) was found between NH and EH (Fig. 5). In order to distinguish the impact of *D. angustifolia* encroaching, we also cultivated the difference among DA and native plots (NS, NH). It is worth noting that Da, NS and NH were significantly different (*P* = 0.002), while there was no significant difference (*P* = 0.265) among DA and encroaching plots (ES, EH). Soil samples from *D. angustifolia* sites and herbaceous sites had similar microbial community structures. We conducted the interdependence between 16 S rDNA transcript abundance of individual phylotypes and single environmental factors using redundancy analysis (RDA, Fig. 6). Of all the environmental variables tested, TN showed the highest correlation with community composition (*P* = 0.009, Table S8). Other factors, such as moisture, pH, MBN and also showed a high (*P* < 0.05) correlation with bacterial community composition (Table S8).

## Discussion

In this work, we present new insights into a native species as an invader from birch forests encroaching into different types of plant communities in the alpine tundra. Our results show that *D. angustifolia* encroaching significantly increases soil nutrient levels (Table 1), which is in agreement with those of previous studies^47^,^48^. The increase range was different between shrub and herbaceous soil samples, which is in line with our hypothesis predicting that the two vegetation types differently responded to encroaching. In our studies, fast growing herbaceous species are often regarded as having higher quantities of litter fall than low shrub species^49^,^50^, and litter of invasive species decomposed more rapidly than that of native species, which might be associated with the higher litter quality of the invasive species^47^,^51^. This can explain the increase in total and available nutrients in soil from *D. angustifolia* encroached sites. Indeed, TOC was strongly correlated to litter layer (Fig. 2D). A small fraction of the nutrients that constitute fungal mycelia may be retained in the soil and contribute to C storage^52^. The amounts of chitin and protein in fungal cell walls are relatively high, and their biopolymers thus represent an important source of both C and nitrogen (N)^20^,^53^,^54^. Correlation analyses indicated that this was an important soil attribute, increasing soil nutrients. As a result of invasion, soil properties of different plant communities were highly variable.

Similar to total nutrients, available nutrients in *D. angustifolia* encroaching sites were increased in the two vegetation types, with higher values in herbaceous than in shrub soil samples (Table 1, Fig. S1). The changes in available nutrients were most likely due to *D. angustifolia* encroaching, significantly increasing soil enzymatic activities. Soil enzymes play an essential role in catalyzing reactions necessary for organic matter decomposition and nutrient cycling^55^,^56^. The activities of soil enzymes are often used as indicators of soil quality, mainly because of their rapid responses to temporary soil changes caused by management and environmental factors^57^,^58^. Our results show that soil nutrients are strongly correlated with soil enzyme activities, indicating that *D. angustifolia* encroaching accelerates decomposition of soil organic matter and enhances mineralization rates of organic N and P. In a similar study, *Zong* (2006) suggested that *D. angustifolia* can effectively occupy the above-ground space by increasing the number of tillers and efficiently using nitrogen, thereby increasing growth^5^. The above mentioned results indicate that *D. angustifolia* encroaching in alpine tundra creates conditions that favor encroaching in new areas, increasing dominance over native plants.

Exotic plants often change ecosystem functions and establish positive plant-soil feedback mechanisms, thereby promoting their dominance^59^. Hereby, the N cycle plays an important role in successful plant invasion^60^,^61^. As expected, *D. angustifolia* altered rhizosphere soil N-cycle dynamics, but these changes varied greatly. The two key findings of our study are: (1) microbially mediated N cycling differed significantly between the two vegetation types, and function genes were influenced by *D. angustifolia*; (2) the change from native sites to encroaching sites, the change was trend to *D. angustifolia* rhizosphere soil micro-ecology. Native rhizosphere soil responded not only to *D. angustifolia* encroachment, but also to different vegetation types.

Soil properties significantly influence microbially mediated N cycling^29^,^62^. In our case, the correlation between soil properties and abundance function genes in two vegetation types was different (Table S4). In herbaceous soil samples, AOA was positively correlated with TOC, TP, and C/N and negative correlated with TK, while shrub soil samples had strong negative correlations with available nutrients (AN, AK). Significant correlations between AOA abundance and organic C, total N, and available N observed in the present study support the idea that C and N conditions may be a key factor affecting the population size of AOA in acidic soil^28^,^63^,^64^,^65^. Ammonia-oxidizing bacteria (AOB) may respond more strongly than ammonia-oxidizing archaea to increased N availability^66^,^67^. However, in our study, this was not the case, probably because ammonia-oxidizing archaea accounted for a large proportion of ammonia oxidizers in our samples. The function of N-fixing genes is to transform N_2_ to 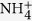 and 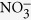, however, we found no correlation between N-fixing gene abundance and 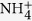 or 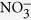. This result was to be expected, since gene presence is not necessarily related to gene activity^68^. Similarly, the level of detection of denitrification genes may be related to the low 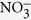 concentrations in the soils. Our analysis revealed that denitrification gene abundance was correlated with soil 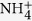 and microbial activity. Similar studies have reported that N-cycle functional genes were contingent on local site characteristics such as soil available nitrogen, soil temperature, and site latitude^68^,^69^,^70^. This suggests that invasive plants, native vegetation types, and local site soil environments were the main factors affecting microbially mediated soil N cycling dynamics.

Plant invasion theory has developed a broad number of hypotheses to explain the success of invasive plants^71^, such as the ability of invaders to alter belowground diversity and processes^38^,^72^. Previously, we suspected native soil microbial community structure to be altered by *D. angustifolia* encroaching, and relevant researches have reported that soil bacteria and fungi were greatly altered by invasive plants^34^. However, we found that *D. angustifolia* encroaching altered shrub soil microbial communities, but had no impact on herbaceous species.

A single invader exhibits negative, positive, or neutral impacts on native diversity, depending on specific locations^42^. We used ANOSIM to elucidate a major mechanism of plant invasion success by comparing the relative abundance of microbial species. The nitrogen-fixing microbes *Devosia (Rhizobiales*) and *Gemmatimonas (Gemmatimonadetes*) showed relative abundances 6.2, and 2.0 times greater in invaded than in native soil samples. These findings are in agreement with those of previous studies reporting that plant invasions are associated with changes of the nitrogen cycle^73^,^74^. Nitrogen-fixing bacterial communities are an important indicator of change in plant invasions^31^. Bacteria of the genus *Conexibacter* were commonly identified as pathogenic bacteria, with abundances decreasing from encroaching to native plots in shrub sites. Soil communities from the invaded range were less pathogenic than from the native range^75^. Our results support the hypotheses that invasive species have less natural enemies in their new range, resulting in competitive advantages over native species^76^,^77^.

Bray-Curtis distance was used to show the beta diversity of the interaction of native and invasion status. The field results show a new phenomenon of bacterial community structure for invasion, i.e. that the bacterial community structure has been changed to suit the requirements of *D. angustifolia*, thereby enabling encroaching. *D. angustifolia* encroaching did not change the herbaceous soil bacterial community structure. This finding contradicts previous studies stating that invasive plants significantly influence native soil bacterial communities^78^,^79^. *D. angustifolia* is a native invasive species in higher altitudes in the Changbai Mountain and has similar life traits as other native herbaceous plants, enabling encroachment. Although there are no studies about which vegetation types were first encroached by *D. angustifolia*, our results still help to fill a major gap in understanding plant invaders and the mechanisms of invasion success. In contrast, *D. angustifolia* encroaching in shrub plant communities significantly changed soil bacterial community structure, and the invaded plots were similar to *D. angustifolia* plots. Our results are consistent with the idea that plant invaders shape belowground communities and establish positively feedback mechanisms^79^. Vegetation types and soil factors such as pH, moisture, and organic matter content significantly influence microbial communities in forest or tundra soils^80^,^81^,^82^. *D. angustifolia* encroaching changed soil properties and indeed plants can determine carbon and nitrogen source and alter soil physical and chemical environment^81^,^82^,^83^,^84^,^85^. Our results show that the composition of the bacterial community was closely correlated with soil TN, moisture, pH and MBN, with agrees with recent studies stating that soil variables are associated with nitrogen transformations and significantly determine bacterial community structure among four vegetation types in the Arctic tundra^86^. *Shen et al*. also suggested soil pH usually the best predictor of variation in microbial diversity^81^. Our results show that the influence of elevation factor on microbial community was not significant. It is largely due to the narrow elevation range and low variance in elevation among treatments. Based on these results, we suggest that microbial community structure is dependent on native vegetation characteristics, and plant species may play a potential role in shaping the rhizosphere microbiome. Invasive plants have therefore a strong ability to disturb the balance of microbial communities and establish new community structures.

As a native invasive species, *D. angustifolia* was better adapted to the new range than non-native species, but encroachment still severely decreased biodiversity levels in the alpine tundra. It should be noticed that dramatic changes in shrub abundance in tundra ecosystems could result in significant alterations of the global carbon cycle^87^. Large areas of shrub degradation occurred in the western slopes of the alpine tundra in the Changbai Mountain^88^. Meanwhile, the results from this study reveal that minor changes in soil microbial communities can cause the decline of the herb community diversity, indicating that herbaceous plant communities may be less resistant to invasion than woody plant communities. Continued degradation of native species in this area will further threat the alpine tundra ecosystem.

## Conclusion

In order to assess the response of rhizosphere soil microbial to *D. angustifolia* encroaching into alpine tundra, we conducted experiments for two vegetation types (shrub and herbaceous). Our results show that encroachment resulted in significant increases of soil nutrients and enzyme activities. *D. angustifolia* was able to alter micorbially mediated nutrient cycling and shrub soil bacterial communities to be suitable for growth, but did not alter the herbaceous soil bacterial communities. The two vegetation community rhizosphere soils responded differently to encroaching. Regardless of the vegetation community, *D. angustifolia* could effectively replace native species. There was a consequent decline in biodiversity, especially shrub degradation. Our research emphasizes that *D. angustifolia* has strong adaptation and expanding abilities by changing rhizosphere soil microbial ecology in alpine tundra. Further encroaching in this area will have serious negative impacts on the tundra.

## Material and Methods

### Study area

Sampling sites were established at the west slope of the Changbai Mountain, located in the Changbai Mountain National Nature Reserve (41°41′49″–42°25′18″N, 127°42′55″–128°16′48″E). The Changbai Mountain have clearly defined vertical vegetation zones: deciduous broad-leaved forest (below 500 m), mixed deciduous broad-leaved/conifer forest (500–1,100 m), dark coniferous forest (1,100–1,700 m); *Betula ermine* forest (1,700–1,950 m), and tundra (>1950 m)^89^. Our study area is located on the western slope of alpine tundra (41°53′–42°04′N, 127°57′–128°11′E, 2,100–2,200 m), where *D. angustifolia* expansion occurred. The climate in the alpine tundra is characterized by low temperatures, heavy precipitation, and a short growing season. Mean annual temperatures in the growing season (June–September) range from 3 to 7 °C. At high altitudes above sea level, annual precipitation exceeds 1,400 mm^90^. Corresponding to the tundra climate, common plants are low stature or prostrate shrubs represented by *D. octopetala var. asiatica*, evergreen shrubs represented by *R. chrysanthum*, deciduous shrubs represented by *Vacciniumu liginosum L*., and tussocks dominated by *Ligularia jamesii (Hemsl.) Kom, Trollius chinensis, Rhodiola cretinii, Sanguisorba tenuifolia var., etc* refs 88 and 91. In this study, we focused on the responses to *D. angustifolia* encroaching of the two dominant plant types, shrubs and herbaceous plants.

### Experimental design

Treatments consisted of *D. angustifolia* communities (DA), native shrub communities (NS), *D. angustifolia* encroaching into shrub (ES), native herbaceous communities (NH), and *D. angustifolia* encroaching into herbaceous communities (EH). *D. angustifolia* sites had less than 5% coverage of other herbaceous plants, and no shrubs were present. In NS treatments, herbaceous plant cover was below 5%, while NH treatments had a *D. angustifolia* coverage below 3%, ES and EH plots had a high *D. angustifolia* coverage (45~60%). Fieldwork was carried out in July 2014. To quantify the invasion impacts of *D. angustifolia* on the native soil function and plant species richness across its entire encroachment range, we selected 16 sampling locations, six of which were mainly colonized by shrubs, five by herbaceous species, and five mainly by *D. angustifolia*. It should be noted that *D. angustifolia* was the only encroachment species in these sites, enabling cause-effect analyses. We compared *D. angustifolia*, native, and encroaching plots at each sampling site. Thus, each pair consisted of encroachment and nearby native plots.

The sites fulfilled the following conditions: (1) *D. angustifolia* generally formed pure stands, having wellestablished and still expanding populations of the target species surrounded by native uninvaded vegetation; (2) Site selection aimed at minimising the probability of differences existing prior to the invasion event. To that end, encroaching and control native plots within a site were in the same topographic situation and had the same soil texture^92^. Moreover, the native control plots were located as close as possible to the front of expansion of the invader. Therefore, systematic differences between encroaching and native soils, if any, can reasonably be ascribed to the contrasted plant cover^48^. Specifically, we surveyed three 1 × 1 m *D. angustifolia*, encroachment, and corresponding native plots. In total, 81 *D. angustifolia*, encroachment, and native plots were surveyed and sampled. We recorded total plant species number and their coverage in all plots. In each plot, four 20 cm × 20 cm subplots were set up for measuring growth and plant harvest.

### Soil sampling

In July 2014, soil samples were collected respectively DA, NS, ES, NH, and EH plots of the 27 sampling regions (see Table S1). In each block, three plots of 1 m × 1 m were randomly selected, aboveground plants and the litter layer were carefully removed. Plant-impacted soil samples were collected by centering a one-inch diameter soil corer around the desired plant and coring to a depth of approximately 10 cm. Each sampled plant was surrounded by plants of the same species as nearest neighbors, and most of these plants grow in relatively high densities, especially *Ligularia jamesii, Sanguisorba tenuifolia var, Rhododendron aureum Georgi,etc.* and, thus, likely have a large impact on the surrounding soil. Therefore, the soil from each core is operationally defined as ‘rhizosphere soil’^93^. About 500 g of soil were extracted at five randomly selected locations. The collected samples from the same block of the same sampling region were mixed to obtain 27 composite soil samples, representing *D. angustifolia*, NS, ES, NH, and EH of each sampling region.

After collection, the samples were immediately refrigerated at 4 °C and transported to the laboratory within 4 h of collection for further processing. Samples were then passed through 2 mm mesh sieve to remove coarse roots, organic debris, and small stones, and each composite sample was assigned into two groups: one group was stored at 4 °C for chemical analyses and the other one at −80 °C for microbial community analysis.

### Analysis of soil characteristics and plant communities

Soil moisture was determined after drying at 105 °C; soil pH was measured on a 1:10 (w/v) ratio in distilled water using a pH meter; soil organic matter was determined by dichromate oxidation with external heat and titration with ferrous ammonium sulphate^94^. Soil TN was determined by semimicro-Kjeldahl (KDY-9820) digestion^95^. Soil AN was measured using the Illinois soil nitrogen test diffusion method^96^. Soil TP was determined colorimetrically using the molybdate method^97^. Soil available phosphorus (AP) was determined colorimetrically based on the Olsen method^98^. Soil TK was extracted by incubation with sodium hydroxide, and soil available potassium (AK) was extracted by incubation with 1.0 mol L^−1^ ammonium acetate for 0.5 h, followed by filtration^96^. Microbial biomass carbon (MBC) and microbial biomass nitrogen (MBN) were determined using the chloroform fumigation extraction method^99^. Soil nitrate nitrogen (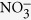
–N) and ammonium nitrogen (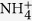
–N) were extracted from 15 g fresh soil with 2 M KCl (soil: extract/1:5) and analyzed using a flow-injection autoanalyzer (Skalar San^++^, Netherlands). Phosphatase activity was measured using the modified method of Schinner and von Mersi^100^. Soil catalase activity was measured using the 0.1N KMnO_4_ titration method^101^. Urease activity was determined according to Klose and Tabatabai^102^. Invertase activity was assayed using the 3,5-dinitrosalicylic acid technique^103^. To assess the effect of *D. angustifolia* encroaching and various treatments on community diversity, we measured the biodiversity of the plant communities. Plant species richness, per cent cover by individual species, and thickness of litter cover were measured by visual estimation in gridded 1 m^2^ frames in each treatment at the peak of the growing season. Aboveground biomass was collected, oven-dried at 85 °C for 48 hours, and weighed.

### Real-time PCR and 16SrDNA

The freeze-dried soil samples (0.3 g) were used for DNA extraction with the Power Soil DNA Isolation Kit (MoBio), according to the manufacturer’s protocol. Extracted DNA was then purified using the GV-High-Efficiency Agarose Gel DNA Purification Kit, DNA concentrations were determined using the Qubit quantification platform with Quant-iT™ ESDNA BR Assay Kit (Invitrogen). DNA was diluted to 10 ng ml^−1^ and stored at −80 °C prior to molecular analysis.

The abundances of genes (*nifH*, archaeal *amoA*, bacterial *amoA, nosZ*) encoding the key enzymes for biological N cycling were quantified by real-time PCR (IBA7500) for all soil samples. These methods were modified from their original descriptions (references following primers) (details see Table S2). Gel-purified PCR products from a common DNA mixture (equal amounts of DNA from all samples) were used to prepare sample-derived quantification standards^104^. The copy number of genes in each standard was calculated by DNA concentration (ng · ul^−1^, measured by Qubit) divided by average molecular weight (PCR product length and GC content was obtained from barcoded-pyrosequencing results) of the amplified gene fragment^63^.

The 16SrDNA high-throughput sequencing was performed by the Realbio Genomics Institute (Shanghai, China) using the Illumina MiSeq platform. The 16S V3-V4 region was amplified using the primers U341F (ACT CCT ACG GGA GGC AGC AG) and U806R (GGA CTA CHV GGG TWT CTA AT). The raw data were then subjected to a quality control procedure using UPARSE^105^. The qualified reads were clustered to generate operational taxonomic units (OTUs) at the 97% similarity level using USEARCH^106^. A representative sequence for each OTU was assigned to a taxonomic level in the RDP database by the RDP classifier^107^.

### Statistical analysis

Statistical analyses were performed in the corrplot package in R. For soil and plant parameters, One-Way analysis of variance (ANOVA) was used to determine differences among DA, native (NS,NH) and invaded(ES,EH) plant communities. The correlations between the abundance of N-cycling functional genes, diversity metrics, and thickness of the litter layer were correlated with soil characteristics using Pearson correlations. Statistical significance was defined as *P* < 0.05.

In QIIME, sequences were subsampled to an even depth of 20067 reads prior to estimating relative taxon abundances and running diversity analyses. Alpha diversity was measured using Chao1, observed species, Shannon, Simpson, goods coverage and PD whole tree. Treatment differences among these metrics were assessed using Wilcox. test and Kruskal. test. Wlcox. test was used to determine differences among native and encroaching soil communities, Kruskal. test determined differences among *D. angustifolia*, native, and encroaching soil communities. Beta diversity used ordinated by non-metric multidimensional scaling (NMDS) using the vegan package of R v.3.1.1 project (distance measure: Bray-Curtis)^108^. Analysis of similarities (anosim, permutations = 999) was used to evaluate the null hypothesis of no difference between groups (distance measure: Bray-Curtis). To determine differences in the microbial communities represented by DA, NS, ES, NH and EH, every isolate in the OTU was considered to be identical to the representative sequence of the OTU. In addition the redundancy analysis (RDA) was performed. RDA is an extension of principal component analysis in which the main components are constrained to be linear combinations of the environmental variables. To determine the environmental factors that significantly correlated with community composition (abundance of OTUs) we used function envfit (permu = 999) using vegan package of R v.3.1.1 project.

## Additional Information

**How to cite this article**: Lin, L. *et al*. Response of rhizosphere soil microbial to *Deyeuxia angustifolia* encroaching in two different vegetation communities in alpine tundra. *Sci. Rep.* **7**, 43150; doi: 10.1038/srep43150 (2017).

**Publisher's note:** Springer Nature remains neutral with regard to jurisdictional claims in published maps and institutional affiliations.

## Supplementary Material

Supplementary Information

## Figures and Tables

**Table 1:** Soil variables (means ± standard deviation) from five sites *D. angustifolia*(DA), native shrub sites(NS), encroaching shrub sites (ES), native herbaceous sites (NH) and encroaching with herbaceous sites (EH) (*n* = 5).

	DA	NS	ES	NH	EH
TN(g/kg)	9.3 ± 0.5d	11.9 ± 0.6b	12.4 ± 0.5a	10.3 ± 0.3c	10.5 ± 0.4c
TC(g/kg)	133.7 ± 5.9c	166.5 ± 8.3b	206.9 ± 6.7a	121.3 ± 5.7c	132.8 ± 5.6c
TP(mg/kg)	530.6 ± 12.0b	422.7 ± 10.9c	518.1 ± 12.3b	354.5 ± 9.4d	560.9 ± 11.7a
TK(g/kg)	9.8 ± 0.3d	12.9 ± 0.3b	11.9 ± 0.3c	16.0 ± 0.4a	12.5 ± 0.4bc
AN(mg/kg)	580.8 ± 11.2c	708.4 ± 12.9b	778.9 ± 11.6a	528.1 ± 13.9d	784.8 ± 13.3a
AP(mg/kg)	18.8 ± 0.6a	14.5 ± 0.8c	16.1 ± 0.8b	8.5 ± 0.8d	14.0 ± 0.5c
AK(mg/kg)	196.9 ± 13.8c	218.1 ± 9.8b	248.4 ± 7.8a	89.9 ± 6.2e	126.5 ± 9.0d
MBC(mg/kg)	1013.0 ± 73.0c	766.4 ± 59.4e	909.9 ± 61.8d	1435.9 ± 43.4b	1600.7 ± 61.9a
MBN(mg/kg)	203.51 ± 13.8c	151.6 ± 10.5d	191.0 ± 5.4c	351.5 ± 6.5b	405.6 ± 17.9a
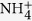 (mg/kg)	181.1 ± 7.2b	209.6 ± 4.9a	144.8 ± 5.8d	169.0 ± 6.4bc	157.2 ± 11.4 cd
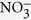 (mg/kg)	28.2 ± 0.9c	48.1 ± 2.1b	35.1 ± 1d	61.8 ± 1.6a	62.0 ± 2.2a
C/N(mg/kg)	13.1 ± 0.5bc	14.0 ± 1.4b	16.9 ± 1.3a	12.0 ± 0.2bc	12.7 ± 0.2c
Urease(mg/kg)	31.3 ± 1.1e	36.9 ± 0.7b	39.8 ± 1.0a	33.0 ± 0.7d	35.2 ± 0.6c
Sucrase(mg/kg)	23.7 ± 0.8c	32.3 ± 2.6a	34.8 ± 2.3a	23.1 ± 2.1c	28.3 ± 2.1b
ACP(mg/kg)	10.8 ± 0.6a	10.4 ± 0.7a	10.8 ± 0.5a	7.7 ± 0.5b	10.5 ± 0.6a
Catalase(mg/kg)	25.0 ± 1.0c	33.3 ± 1.3a	33.9 ± 0.8a	29.3 ± 1.2b	32.1 ± 1.0a
pH	4.7 ± 0.1c	4.4 ± 0.1d	4.8 ± 0.4b	5.0 ± 0.2a	4.7 ± 0.2c
Moisture(%)	44.2 ± 1.0d	59.9 ± 1.3a	47.3 ± 1.2c	46.3 ± 2.3 cd	50.9 ± 1.5b

The lower case letters ‘a’, ‘b’, ‘c’ and ‘d’ indicate contrasts that are significantly different (*P* < 0.05) among different treatment.

**Table 2:** Genera with a greater relative abundance associated with native sites and encroaching sites under shrub and herbaceous level treatment to have a significant effect based on Indicator Species Analysis (*P*-value < 0.05).

Types	Phylum	Class	Order	Family	Genus	N (%)	E (%)	Up/down regulation	*P*
S	Proteobacteria	Alphaproteobacteria	Rhizobiales	Hyphomicrobiaceae	Devosia	0.019	0.117	+	0.025
	Proteobacteria	Gammaproteobacteria	Enterobacteriales	Enterobacteriaceae	Dokdonella	0.004	0.048	+	0.026
	Proteobacteria	Deltaproteobacteria	Myxococcales	Phaselicystidaceae	Phaselicystis	0.023	0.103	+	0.008
	Proteobacteria	Alphaproteobacteria	Rhodobacterales	Rhodobacteraceae	Rhodobacter	0	0.053	+	0.044
	Actinobacteria	Actinobacteria	Solirubrobacterales	Conexibacteraceae	Conexibacter	1.196	0.874	−	0.040
	Actinobacteria	Actinobacteria	Actinomycetales	Nakamurellaceae	Nakamurella	0.006	0.098	+	0.010
	Actinobacteria	Actinobacteria	Actinomycetales	Streptosporangiaceae	Streptosporangium	0	0.015	+	0.042
	Gemmatimonadetes	Gemmatimonadetes	Gemmatimonadales	Gemmatimonadaceae	Gemmatimonas	0.363	0.727	+	0.018
	Parcubacteria	/	/	/	Parcubacteria	0.024	0.009	−	0.042
	Verrucomicrobia	Subdivision3	/	/	Subdivision3	0.059	0.017	−	0.050
H	Proteobacteria	Alphaproteobacteria	Caulobacterales	Caulobacteraceae	Asticcacaulis	0.003	0.010	+	0.045
	Acidobacteria	Acidobacteria	/	/	Granulicella	0.629	1.124	+	0.017

S indicates shrub level and H indicates herbaceous level.
