# Supplementary material for: Response of rhizosphere soil microbial to Deyeuxia angustifolia encroaching in two different vegetation communities in alpine tundra
Source: Sci Rep. 2017 Feb 21;7:43150. doi: 10.1038/srep43150 (PMC5318906; doi:10.1038/srep43150)
Supplement: Supplementary Information [file srep43150-s1.pdf]

Response of rhizosphere soil microbial to *Deyeuxia angustifolia* encroaching in two different vegetation communities in alpine tundra

Li Lin [1,3], Xing Ming [1,3], Lv Jianwei[2], Wang Xiaolong [1,3] , Chen Xia[1, 3*]

[1]*National & Local United Engineering Laboratory for Chinese Herbal Medicine Breeding and Cultivation, Jilin University, Changchun 130112, China.*

[2]*Huhhot Vocational college, Huhht, Inner Mongolia 010051, China.*

[3]*School of Life Science, Jilin University, Changchun 130012, China.*

[*]*For correspondence. E-mail address: chenxiajlu@163.com*

TableS1. Sampling sites, geographical location and dominant species that use plant coverage (%) to indicate.

Types	Altitude(m)	Latitude	Longitude	Dominant species & Coverage (%)
NH	2182	41°59′25.57″	128°1′0.23″	*Ligularia jamesii (Hemsl.) Kom.* 43%, *Sanguisorba sitchensis*46%, *Veratrum nigrum L.* 7%
EH	2182	41°59′25.57″	128°1′0.23″	*D. angustifolia* 40%, *Ligularia jamesii (Hemsl.) Kom*. 22%, *Sanguisorba sitchensis* 26%
NS	2182	41°59′25.57″	128°1′0.23″	*Rhododendron aureum* Georgi 60%,*Vaccinium uliginosum* Linn. 28%
ES	2182	41°59′25.57″	128°1′0.23″	*D. angustifolia 35%, Rhododendron aureum* Georgi 35%, *Vaccinium uliginosum Linn.*25%
DA	2182	41°59′25.57″	128°1′0.23″	*D. angustifolia* 92%
NS	2178	41°59′20.99″	128°0′52.65″	*Vaccinium uliginosum Linn.*68%, *Phyllodoce caerulea (L.) Bab.*24%
ES	2178	41°59′20.99″	128°0′52.65″	*D. angustifolia* 49%, *Vaccinium uliginosum Linn.*28%, *Phyllodoce caerulea (L.) Bab.* 15%
NS	2178	41°59′20.99″	128°0′52.65″	*Rhododendron aureum Georgi* 83%, *Vaccinium uliginosum Linn.*8%
ES	2178	41°59′20.99″	128°0′52.65″	*D. angustifolia* 49%, *Rhododendron aureum Georgi* 42%
DA	2178	41°59′20.99″	128°0′52.65″	*D. angustifolia* 90%
NS	2143	41°59′25.02″	128°0′45.68″	*Rhododendron aureum Georgi* 95%, *Vaccinium uliginosum Linn.*3%
ES	2143	41°59′25.02″	128°0′45.68″	*D. angustifolia* 45%, *Rhododendron aureum Georgi* 51%
NS	2143	41°59′25.02″	128°0′45.68″	*Vaccinium uliginosum Linn.*92%
ES	2143	41°59′25.02″	128°0′45.68″	*D. angustifolia* 55%, *Vaccinium uliginosum Linn.*36%
NH	2142	41°59′25.02″	128°0′45.68″	*Ligularia jamesii (Hemsl.) Kom*. 82%, *Trollius chinensis Bunge* 7%, *Veratrum nigrum L.* 5%
EH	2143	41°59′25.02″	128°0′45.68″	*D. angustifolia* 45%, *Ligularia jamesii (Hemsl.) Kom*42%, *Trollius chinensis Bunge* 6%
DA	2143	41 °59′25.02″	128°0′45.68″	*D. angustifolia* 93%
NS	2034	41°59′20.92″	128°0′9″	*Phyllodoce caerulea (L.) Bab.*34%, *Rhododendron aureum Georgi* 52%, *Vaccinium uliginosum Linn.*9%
ES	2034	41°59′20.92″	128°0′9″	*D. angustifolia* 47%, *Phyllodoce caerulea (L.) Bab*. 15%,*Rhododendron aureum Georgi* 23%, *Vaccinium uliginosum Linn.*7%
NH	2034	41°59′20.92″	128°0′9″	*Sanguisorba sitchensis* 60%, *Trollius chinensis Bunge* 22%, *Aconitum monanthum Nakai*8%, *Veratrum nigrum* L. 5%
EH	2034	41°59′20.92″	128°0′9″	*D. angustifolia* 55%,*Sanguisorba sitchensis* 30%,*Trollius chinensis Bunge* 8%,*Aconitum monanthum Nakai* 5%
NH	2034	41°59′20.92″	128°0′9″	*Sanguisorba sitchensis* 30%, *Aconitum monanthum Nakai* 21%, *Veratrum nigrum L.* 15%, *Trollius chinensis Bunge* 18%, *Ligularia jamesii (Hemsl.) Kom* 10%
EH	2034	41°59′20.92″	128°0′9″	*D. angustifolia* 42%,*Sanguisorba sitchensis* 18%, *Aconitum monanthum Nakai* 12%, *Trollius chinensis Bunge* 8%, *Veratrum nigrum L.* 5%
DA	2034	41°59′20.92″	128°0′9″	*D. angustifolia 90%,Veratrum nigrum* L. 5%
NH	2026	41°59′15.72″	128°0′6.29″	*Trollius chinensis Bunge* 35%, *Sanguisorba sitchensis* 30%, *Aconitum monanthum Nakai* 12% *Veratrum nigrum L.* 8%, *Ligularia jamesii (Hemsl.) Kom* 12%
EH	2026	41°59′15.72″	128°0′6.29″	*Trollius chinensis Bunge* 25%, *Sanguisorba sitchensis* 9%, *Aconitum monanthum Nakai* 7%, *Veratrum nigrum L.* 4%
DA	2026	41°59′15.72″	128°0′6.29″	*D. angustifolia* 83%, *Trollius chinensis Bunge* 6%

TableS2. Primers and real-time PCR conditions used in this experiment.

function genes	primers		real-time PCR protocol
nifH	PolF	5′-TG CGAY CCS AAR GCB GAC TC-3′	94 ℃ for 5 min; 40 cycles of 94 ℃ for 30s, 57 ℃ for 45s(54 ℃ for *nifH* gene, 60 ℃ for AOB), 72 ℃ for 1 min.
	PolR	5′-ATS GCC ATC ATY TCR CCG GA-3′	
AOA	Arch-amoAF	5′-STA ATG GTC TGG CTT AGA CG-3′	
	Arch-amoAR	5′-GCG GCC ATC CAT CTG TAT GT-3′	
AOB	amoA-1F	5′-GGG GTT TCT ACT GGT GGT-3′	
	amoA-2R	5′-CCC CTC KGS AAA GCC TTC TTC-3′	
nosZ	nosZ-F	5′-CGY TGT TCM TCG ACA GCC AG-3′	
	nosZ 1622R	5′-CGS ACC TTS TTG CCS TYG CG-3′	

TableS3. Pearson correlation coefficient (*r*) between soil properties and enzymatic activities, Asterisk indicates significant differences *indicate significant (*P*<0.05) effects, ** indicate significant (*P*<0.01) effects.

	TN	TOC	TP	TK	NO_3^-	NH_4^+	Moisture	pH	MBC	MBN	AN	AP	AK	C/N
Urease	0.616**	0.715**	0.329*	-0.288	-0.153	0.082	0.567**	-0.336	0.016	-0.076	0.515**	0.290	0.422*	0.606**
Sucrase	0.484**	0.714**	0.177	-0.260	-0.278	0.275	0.402*	-0.168	0.094	-0.318	0.317	0.084	0.324*	0.683**
ACP	0.062	0.242	0.490**	-0.086	-0.030	0.240	0.310	-0.409	0.037	-0.087	0.297	0.651**	0.543**	0.233
Catalase	0.748**	0.731**	0.387*	0.034	0.013	0.305	0.592**	-0.244	0.262	-0.100	0.440*	0.336*	0.413*	0.561**

TableS4. Pearson correlation coefficient (*r*) between N cycling function genes abundance and soil properties, just list significant (*P*<0.05) correlation. Bolded values indicate significant (*P*<0.05) effects.

	AOB		AOA		*nosZ*		*nifH*	
	r	*P*	*r*	*P*	*r*	*P*	*r*	*P*
**Total**	**n=27**							
NO_3^-	**-0.404**	**0.036**	**0.451**	**0.018**	-0.073	0.719	-0.050	0.806
AP	**-0.410**	**0.040**	-0.061	0.764	0.291	0.141	-0.324	0.099
TP	-0.245	0.218	0.298	0.131	0.039	0.847	**0.461**	**0.016**
NH_4^+	-0.118	0.557	-0.132	0.512	**0.560**	**0.002**	-0.207	0.300
Moisture	0.020	0.922	-0.096	0.634	**0.490**	**0.009**	0.013	0.950
**Herbaceous**	**n=10**							
AP	**-0.636**	**0.048**	-0.240	0.505	-0.341	0.334	-0.463	0.178
TOC	0.249	0.489	**0.833**	**0.003**	0.207	0.506	**0.815**	**0.004**
TP	-0.232	0.518	**0.672**	**0.033**	-0.023	0.950	**0.668**	**0.035**
C/N	0.293	0.412	**0.701**	**0.024**	0.190	0.599	**0.698**	**0.025**
TK	-0.514	0.128	**-0.671**	**0.034**	0.193	0.592	**-0.632**	**0.048**
MBC	-0.425	0.221	-0.213	0.554	**-0.633**	**0.049**	-0.469	0.171
MBN	-0.452	0.190	-0.357	0.311	**-0.665**	**0.036**	-0.542	0.106
Moisture	0.475	0.165	**0.626**	**0.530**	0.456	0.185	**0.633**	**0.037**
**Shrub**	**n=12**							
AN	0.420	0.174	**-0.591**	**0.043**	-0.029	0.928	0.533	0.074
AK	-0.173	0.582	**-0.643**	**0.024**	**0.334**	**0.288**	0.431	0.161
NH_4^+	-0.231	0.471	-0.437	0.155	**0.620**	**0.031**	-0.332	0.291
MBC	-0.240	0.460	-0.143	0.658	**0.841**	**0.001**	0.134	0.679

TableS5. Alpha Diversity Metrics for Native sites (NH, NS), Encroaching sites (EH, ES) in two vegetation types and *D. angustifolia* (DA) bacteria.

	**chao1**	**observed species**	**PD whole tree**	**shannon**	**simpson**	**goods coverage**
NS	1100.42	857.5	54.29	7.86	0.99	0.99
ES	925.80	826.83	48.02	7.40	0.98	0.99
NH	1037.12	865.40	52.10	7.45	0.98	0.99
EH	998.05	834.80	51.02	7.36	0.98	0.99
DA	863.95	747.75	48.38	7.62	0.98	0.99

TableS6. A non-parametric test showed differences between encroaching and native sites in two treatments (NS/ES and NH/EH), compare the microbial diversity *D. angustifolia* to native samples (DA/NS/NH) and to encroaching samples (DA/ES/EH). Bolded values indicate significant (α<0.05) effects.

Alpha name	NS/ES	NH/EH	DA/NS/NH	DA/ES/EH
chao1	**0.004**	0.55	**0.05**	0.09
observed species	0.618	0.31	0.26	0.17
PD whole tree	**0.025**	0.84	0.48	0.36
shannon	**0.037**	0.84	0.70	0.82
simpson	0.113	1.00	0.33	0.76
goods coverage	0.857	0.84	**0.03**	0.22

TableS7. Phylum with a greater relative abundance associated with native and encroaching sites under woody and herbaceous treatment to have a significant effect based on ANOSIM Analysis. Bolded values indicate significant (*P*<0.05) effects.

Phylum	NS/ES	NH/EH	DA/NS/NH	DA/ES/EH
Acidobacteria	0.07	0.43	0.17	0.17
Proteobacteria	**0.05**	**0.05**	0.12	0.66
Actinobacteria	0.11	0.43	0.43	0.15
Bacteroidetes	0.09	0.25	**0.04**	0.58
Chloroflexi	0.61	0.66	0.41	0.71
Candidatus Saccharibacteria	0.69	0.93	0.08	0.20
Planctomycetes	**0.04**	1.00	0.42	0.31
Firmicutes	0.61	0.93	0.35	0.45
Gemmatimonadetes	**0.02**	0.79	**0.04**	0.64
Verrucomicrobia	0.08	0.54	0.51	0.61
Nitrospirae	0.50	1.00	**0.04**	0.63
Latescibacteria	1.00	0.40	**0.05**	0.68
Cyanobacteria/Chloroplast	0.17	0.27	0.31	0.47
Chlamydiae	0.26	0.51	0.61	0.70

TableS8. The correlation between community composition and environmental variables for bacteria. Values in bold indicate significant correlation (*P*<0.05).

**Variable**	***r***	***Pr* (>*r*)**
**TN**	**0.353**	**0.009 ****
**Moisture**	**0.262**	**0.030 ***
**pH**	**0.261**	**0.024 ***
**MBN**	**0.274**	**0.029 ***
MBN	0.204	0.068
AK	0.174	0.096
Elevation	0.153	0.128
TOC	0.157	0.129
NH_4^+	0.100	0.266
AP	0.055	0.520
AN	0.034	0.663
TK	0.026	0.722
NO_3^-	0.022	0.745
TP	0.020	0.794

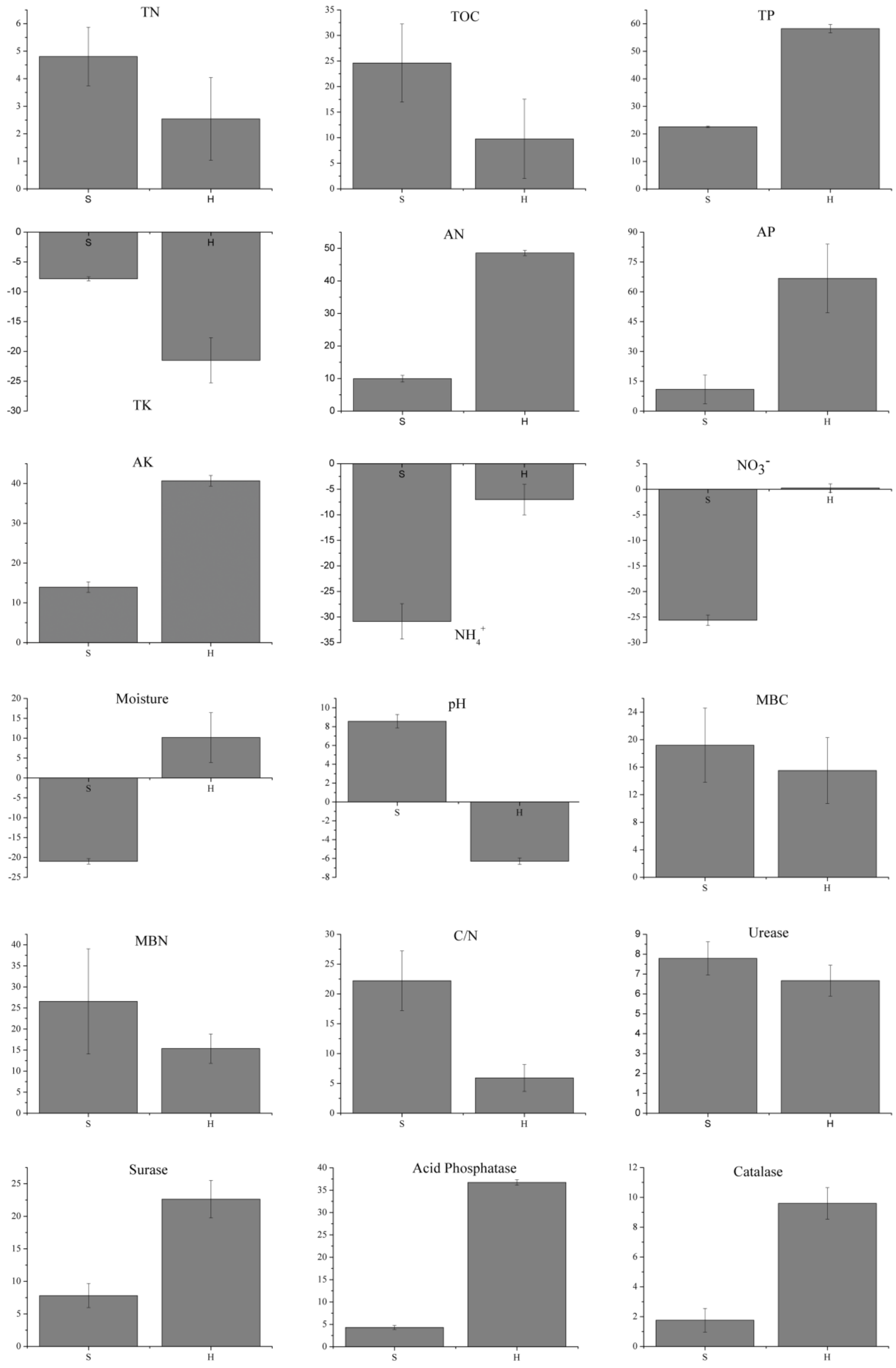

Fig.S1. The effect of *D. angustifolia* encroaching on soil nutrients are shown. Data are pooled over vegetation types and were generated by looking at the percentage loss or gain of a particular attribute *D. angustifolia* encroaching relative to the native control values. Bars are standard errors.

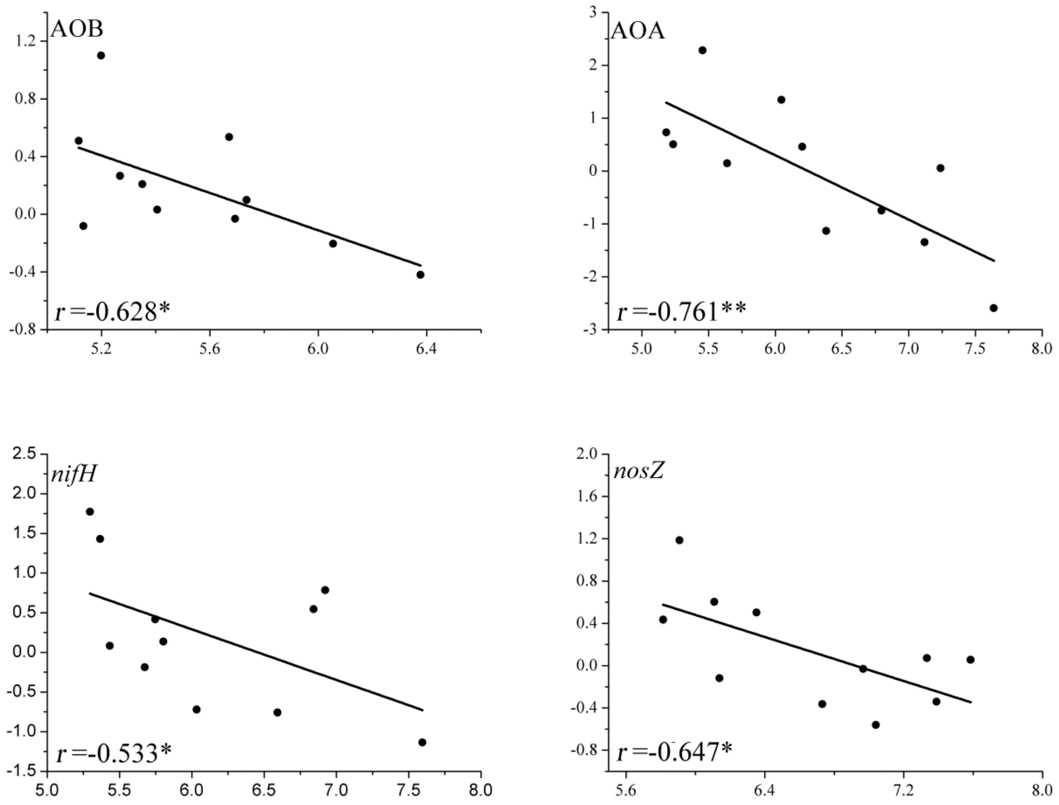

Fig.S2. Impact of invasive species on N-transforming function genes in native plots (*N*). Impact is calculated as the difference between the value of the parameter in encroaching plots (*E*) minus the value of the parameter in native plots (*N*). A positive value of *E*-*N* indicates an increased concentration of that element in the invaded soil Pearson correlation coefficient (*r*) between *N* and *E* -*N*. Significance levels(*P < 0.05, **P < 0.01).

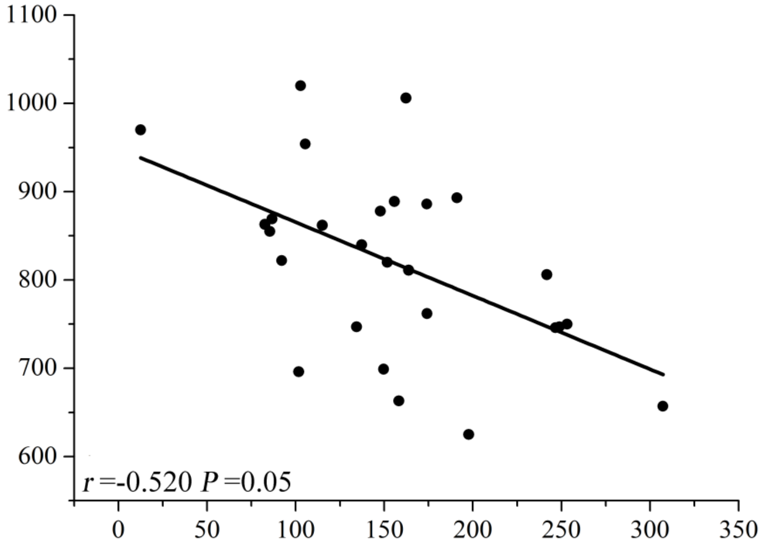

Fig.S3. Pearson correlation coefficient (*r*) between OTU richness and TOC.
